# Discovery of Single Nucleotide Polymorphisms for Resistance to Abnormal Vertical Growth in Macadamia

**DOI:** 10.3389/fpls.2021.756815

**Published:** 2021-12-24

**Authors:** Mohamed Cassim Mohamed Zakeel, Mobashwer Alam, Andrew D. W. Geering, Bruce Topp, Olufemi A. Akinsanmi

**Affiliations:** ^1^Centre for Horticultural Science, Queensland Alliance for Agriculture and Food Innovation, The University of Queensland, Brisbane, QLD, Australia; ^2^Centre for Horticultural Science, Queensland Alliance for Agriculture and Food Innovation, The University of Queensland, Nambour, QLD, Australia

**Keywords:** DArT, markers, polymerase chain reaction, Proteaceae, resistance breeding, tree nut

## Abstract

Abnormal vertical growth (AVG) syndrome is a serious threat to the Australian macadamia industry as it decreases the yield of nuts by as much as 70% per annum. A lack of information on the cause of AVG has hindered the development of an effective disease management strategy. Discovery of genetic markers associated with disease resistance can be used as tool for rapid selection of elite cultivars, hence helps in efficient disease management. Differences in field susceptibility of macadamia cultivars provide an opportunity for discovery of genetic markers that are associated with host resistance. REML mixed model analysis was performed to estimate the AVG rating of 51 cultivars from multiple origins using phenotypic data from 359 trees planted in four sites. Most of the Hawaiian cultivars were found as susceptible, while selections from the Australian macadamia industry breeding program were predominantly resistant. All the cultivars were genotyped for 13,221 DArTseq-based single nucleotide polymorphism (SNP) markers. A bulked sample analysis was performed using 20 genotypes each at the extremes of AVG phenotypic ratings. Ten SNP markers were predicted to be associated with AVG resistance and two arbitrarily selected SNP markers were validated using PCR and Sanger sequencing. Our findings suggest that AVG resistance in the commercial cultivars may be derived from the genomic introgression of *Macadamia tetraphylla* through interspecific hybridization. The results may support marker-assisted selection for macadamia germplasm with AVG resistance.

## Introduction

Macadamia is a recently domesticated, evergreen nut tree that is popular for its cream-colored kernel. Macadamia has wide climatic tolerance, allowing it to be cultivated in many subtropical and frost-free temperate regions of the world including Australia, China, New Zealand, South Africa, and the United States, and in tropical regions in Brazil, Guatemala, Kenya, Malawi, and Vietnam (Hamilton et al., 1983; Supamatee et al., 1992; Xiao et al., 2002; Piza et al., 2006). The genus *Macadamia* (family Proteaceae) includes four species that are endemic to the subtropical rainforests of eastern Australia (Stephenson, 2005; Hardner et al., 2009), but only *M. integrifolia* (smooth-shelled) and *M. tetraphylla* (rough-shelled) are edible (Gross, 1995; Stephenson, 2005). Hybrids of these two species are very common in commercial cultivation (Hardner et al., 2009). The current commercial macadamia cultivars are only one to two generations away from their wild progenitors in the rain forest. Most of these cultivars were not selected based on structured breeding programs, but mainly from open pollinated seedlings focusing on their yield performance (Hardner et al., 2009).

Macadamia is open-pollinated and highly heterozygous, providing opportunities to map candidate gene(s) and quantitative trait loci (QTL) for all types of traits (Peace et al., 2003). Randomly Amplified Microsatellite Fingerprinting (RAMiFi) and RAF markers have been used to define natural species distribution, explore species composition in hybrids, trace pollen flow and the origin of cultivated germplasm (Peace et al., 2002, 2003, 2005). RAMiFi and RAF markers have been used to construct genetic maps and phylogenetic trees of macadamia cultivars (Peace et al., 2001a,^2002^, ^2003^, ^2005^). Simple sequence repeats markers have been used to analyze macadamia pedigree (Schmidt et al., 2006). Based on DNA typing, Peace et al. (2001b) classified macadamia cultivars into seven gene pools (1–7). The gene pools 1 and 2 contain most Hawaiian cultivars, a few Australian cultivars and some cultivars developed in Israel, while gene pools 3 and 4 consisted of Australian cultivars that contain a large proportion of *M. integrifolia* genome (Peace et al., 2001b,^2017^). Most of the Australian hybrid cultivars are within the gene pools 5 and 6, while the gene pool 7 includes hybrids and pure *M. tetraphylla* cultivars selected in Australia and South Africa (Peace et al., 2001b). Hawaiian cultivars have a narrow genetic base and originated from a single chlorotype of plants located at Mooloo and Mt Bauple (Alam et al., 2018; Nock et al., 2019), while Australian cultivars are genetically diverse and have multi-species combinations in their genome (Peace et al., 2017; Alam et al., 2018).

The Australian macadamia industry is threatened by a syndrome known as abnormal vertical growth (AVG), which has an unknown etiology. Recent studies have hypothesized a biotic cause for AVG while dismissing previous hypothesis that AVG is caused by *Bacillus megaterium*, phytoplasmas or a geminivirus (Zakeel et al., 2020, 2021). AVG is characterized by a vigorous upright growth habit with reduced lateral branching, flower production and nut set (O’Farrell, 2011). It takes about 10 years for symptoms of AVG to develop to a point where affected trees can be unambiguously distinguished from healthy trees (O’Farrell et al., 2016). AVG incidence was first reported in 1990s, but its importance was not recognized until recently. None of the elite cultivars were selected for AVG resistance. Currently, the impact of AVG on yield loss is very high (Zakeel et al., 2020), thus it is a major concern to the industry. Using the diversity of existing cultivars can be useful to identify the variability for AVG resistance. Surveys by O’Farrell et al. (2016) and Akinsanmi (2017) have shown that most Hawaiian cultivars such as ‘HAES 344’, ‘HAES 741’ and ‘HAES 246’ are very susceptible to AVG, whereas the Australian cultivars such as ‘A4’ and ‘A16’ are resistant and ‘A268’, which is a progeny of the Hawaiian cultivar ‘HAES 344 and an unknown *M. tetraphylla* pollen parent (Hardner et al., 2009; Peace et al., 2017), is moderately susceptible to AVG. Australian cultivars are often *M. integrifolia* × *M. tetraphylla* hybrids (Peace et al., 2001b; Nock et al., 2019). There is no control option in place for AVG trees, and agronomic management practices rarely improve the yield of severely affected trees (O’Farrell et al., 2016). Replanting of macadamia still poses a risk of reoccurrence of AVG (Akinsanmi, 2017). Information on varietal susceptibility is limited, thus, plant breeding with the objective of selecting individuals with superior performance may offer an effective control option for AVG in Australia.

Genomic selection and marker-assisted selection (MAS) approaches rely on marker–trait associations and are both routinely used for breeding purposes. The indirect selection of AVG resistant markers that confer polymorphisms to AVG from a large number of markers dispersed across the genome may be used to predict or estimate AVG resistance in macadamia germplasm without having an accurate knowledge of where specific genes are located. Genetic markers such as single nucleotide polymorphism (SNP) that covers the entire genome are routinely used for breeding purposes, so that all QTL of interest are in linkage disequilibrium with at least a single marker (Quarrie et al., 1999; Becker et al., 2011; Zila et al., 2014; Chen et al., 2016; Patil et al., 2016; Alam et al., 2018; de Jong et al., 2018). MAS may provide solutions to most problems associated with macadamia breeding for AVG resistance.

Traditional breeding in macadamia takes 8–10 years and is expensive because large areas of land are required and the trees need ongoing maintenance (Topp et al., 2019). The long process for phenotyping can be circumvented using molecular markers (Mohan et al., 1997; Ribaut and Hoisington, 1998; Agarwal et al., 2008; Heffner et al., 2009; Ashraf and Foolad, 2013) and MAS to accurately identify traits of interest including resistance to biotic and abiotic stresses (Schachermayr et al., 1994; Cervera et al., 1996; Quarrie et al., 1999). The ultra-high-throughput diversity array technology (DArT) markers have been used to identify the genetic diversity and DArTseq-based SNP markers were successfully utilized to confirm the genetic identity of macadamia genotypes (Alam et al., 2018). Alam et al. (2018) suggested that DArT platforms are a robust system enabling genomic studies in macadamia. DArTseq-based SNP markers for over 500 commercial macadamia cultivars and 300 accessions of wild germplasm are available (Alam et al., 2018; Mai et al., 2020). Among these cultivars, there are some that are very prone to developing AVG in the field, while others appear to be resistant, even when grown at ‘AVG hotspots’ (Akinsanmi, 2017). A current limitation of the use of these DArT markers in macadamia is that their locations are unknown, as a complete reference genome is currently not available. However, a putative discovery of significant SNP markers for AVG resistance would accelerate MAS in macadamia breeding program, when a well-annotated complete reference genome for macadamia becomes available. Thus, DArTseq based genotyping method has been used for association studies in macadamia (O’Connor et al., 2019, 2020). Anchoring each SNP marker to the genome sequence and genetic mapping would improve its usefulness (Jia et al., 2016) as is used in platforms such as Illumina and Affymetrix (Bayer et al., 2017; Zhao et al., 2020; Soumya et al., 2021).

In order to identify any SNP markers for AVG resistance, we utilized the genotypic data from the DArTseq for macadamia cultivars that had been phenotyped for AVG in commerical orchards. We tested the hypothesis that variation among the macadamia genotypes to AVG severity is associated with certain genetic markers. Thus, we provided insights into the genetic basis of AVG resistance.

## Materials and Methods

### Germplasm Used

Three hundred and fifty-nine macadamia trees representing 51 cultivars were used in this study (Table 1). Mature trees (>10-year-old) were assessed in four commercial macadamia orchards with history of AVG in the southeast Queensland. The cultivars representing a mixture of commercial cultivars with field resistance and susceptibility to AVG, belong to five origins of selection (AES: Australian Early Selection; AMIB: Australian Macadamia Industry Breeding; HAES: Hawaii Agricultural Experiment Station; HVP: Hidden Valley Plantation; and Macadamia Conservation Trust: MCT) (Table 1). The cultivars were selected for commercial cultivation based on agronomic characteristics and yield performance. Based on Peace et al. (2001b) classification, the cultivars used in this study were classified into different gene pools, with nine cultivars in gene pool 1, three cultivars each in gene pool 2 and 6, one cultivar each in gene pool 3 and 4, and four cultivars in gene pool 5, while 30 cultivars had unknown gene pool classification (Table 1).

**TABLE 1:** Macadamia cultivars used in the study for resistance ratings for abnormal vertical growth, their estimated AVG rating and susceptibility or resistance group.

Cultivars	Origin	Gene pool*^a^*	Sites	No. of replicate trees	Estimated AVG rating*^b^*	AVG resistance group
Beaumont	AES	5	B-VT	2	0.17	Susceptible
			W-T	4		
Daddow	AES	4	H-T2	4	0.08	Susceptible
			W-VT	4		
NG8	AES	5	B-VT	6	0	Resistant
			H-T2	4		
			W-VT	4		
Own venture	AES	3	H-T2	4	0.05	Susceptible
			W-VT	4		
1/40B	AMIB	Unknown	W-VT	4	0	Resistant
2/48B	AMIB	Unknown	W-VT	4	0	Resistant
MIV-A (A)	AMIB	Unknown	W-T	4	0	Resistant
MIV-B (B)	AMIB	Unknown	W-T	4	0	Resistant
MIV-C (C)	AMIB	Unknown	W-T	3	0	Resistant
MIV-D (D)	AMIB	Unknown	W-T	2	0	Resistant
MIV-E (E)	AMIB	Unknown	W-T	4	0	Resistant
MIV-F (F)	AMIB	Unknown	W-T	4	0	Resistant
MIV-G (G)	AMIB	Unknown	W-T	4	0	Resistant
MIV-H (H)	AMIB	Unknown	W-T	4	0	Resistant
MIV-I (I)	AMIB	Unknown	W-T	3	0	Resistant
MIV-J (J)	AMIB	Unknown	W-T	4	0	Resistant
MIV-K (K)	AMIB	Unknown	W-T	3	0.11	Susceptible
MIV-L (L)	AMIB	Unknown	W-T	4	0.17	Susceptible
MIV-M (M)	AMIB	Unknown	W-T	4	0	Resistant
MIV-M (N)	AMIB	Unknown	W-T	4	0	Resistant
MIV-O (O)	AMIB	Unknown	W-T	2	0	Resistant
MIV-P (P)	AMIB	Unknown	W-T	4	0	Resistant
MIV-Q (Q)	AMIB	Unknown	W-T	4	0.08	Susceptible
MIV-R (R)	AMIB	Unknown	W-T	4	0	Resistant
MIV-S (S)	AMIB	Unknown	W-T	2	0	Resistant
MIV-T (T)	AMIB	Unknown	W-T	4	0	Resistant
			W-T	4		
A4	HVP	6	H-T2	3	0.05	Susceptible
			W-VT	4		
			B-VT	2		
A16	HVP	6	W-T	4	0	Resistant
			H-T2	4		
			W-VT	4		
A38	HVP	Unknown	H-T2	4	0.15	Susceptible
			W-VT	4		
A199	HVP	6	H-T2	4	0	Resistant
			W-VT	4		
A203	HVP	Unknown	H-T2	4	0	Resistant
			W-VT	4		
A268	HVP	5	B-VT	6	0.04	Susceptible
			W-T	5		
			H-T2	4		
			W-VT	4		
A376	HVP	Unknown	W-T	1	0	Resistant
A403	HVP	Unknown	W-T	3	0	Resistant
			W-T	4		
A422	HVP	Unknown	H-T2	4	0.04	Susceptible
			W-VT	4		
A447	HVP	Unknown	W-T	3	0	Resistant
A538	HVP	Unknown	W-T	4	0	Resistant
			B-VT	2		
HAES246	HAES	1	H-T2	4	0.25	Susceptible
			W-VT	4		
HAES333	HAES	1	W-T	2	0.17	Susceptible
			B-VT	6		
HAES344	HAES	2	W-T	34	0.36	Susceptible
			H-T2	4		
			W-VT	4		
HAES705	HAES	5	W-VT	4	0.25	Susceptible
			B-VT	3		
HAES741	HAES	2	H-T2	4	0.18	Susceptible
			W-VT	4		
HAES772	HAES	2	H-T2	4	0.33	Susceptible
			H-T2	4		
HAES781	HAES	1	W-VT	4	0.43	Susceptible
HAES783	HAES	1	H-T2	4	0.03	Resistant
			W-VT	4		
HAES804	HAES	1	H-T2	4	0.33	Susceptible
			B-VT	2		
HAES814	HAES	1	H-T2	2	0.03	Resistant
			W-VT	4		
HAES816	HAES	1	B-VT	2	0.14	Susceptible
			W-T	4		
			H-T2	4		
			W-VT	4		
HAES842	HAES	1	B-VT	6	0.13	Susceptible
			W-T	4		
			H-T2	4		
			W-VT	4		
HAES849	HAES	1	B-VT	2	0.09	Susceptible
			H-T2	4		
			W-VT	4		
4/7Mc (MCT1)	MCT	Unknown	W-VT	4	0	Resistant

*AES, Australian Early Selection; AMIB, Australian Macadamia Industry Breeding; HAES, Hawaii Agricultural Experiment Station; HVP, Hidden Valley Plantation; MCT, Macadamia Conservation Trust.*

*Mature trees (>10 year-old) were used in this study.*

*^a^Based on the classification by Peace et al. (2001b).*

*^b^Based on restricted maximum likelihood (REML) mixed model.*

### Assessment of Phenotypic Data of Macadamia Cultivars for Resistance to Abnormal Vertical Growth

All the 359 macadamia trees were rated for AVG using the severity rating scale of 0–3 as described by Akinsanmi (2017), where 0 = no AVG (no visible symptoms of AVG); 1 = suspicious AVG (only 1–3 branches inside the canopy show symptoms of AVG; 2 = mild AVG (distinct AVG symptoms on most branches) and 3 = severe AVG (distinct AVG crown appearance with AVG symptoms on lower branches). The panel of cultivars at each site varied, as did the number of replicate trees per cultivar thus, the experimental design was unbalanced structure. Nevertheless, five cultivars (‘HAES 842’, ‘HAES 816’, ‘HAES 344’, ‘A268’ and ‘A16’) were represented at all the four sites (Table 1). The phenotypic AVG severity data were analyzed using a Restricted Maximum Likelihood (REML) mixed model:


$$P_{a\,v\,g}=m+G+S+B+G*S+G*B+S*B+G*S*B+\varepsilon$$

where *P*_*avg*_ is the phenotypic value for AVG, *m, G, S*, *B* and ε represent the mean, cultivar, trial site, block and residual error, respectively. In the mixed model analysis, m, G, S, B, G*S, G*B, S*B, and G*S*B were considered as fixed factors and ε as random. AVG rating (*ER*_*AVG*_) for each cultivar was estimated using the best linear unbiased estimates of *G*. Twenty cultivars with *ER*_*AVG*_ = 0, were selected as extremely resistant group (bulk) and another 20 cultivars with *ER*_*AVG*_ > 0 were selected as extremely susceptible group with no correction for population structure.

### Assessment of Genotypic Data of Macadamia Cultivars for Resistance to Abnormal Vertical Growth

The genotypic data (DArTseq) of the 51 cultivars that were previously sequenced by Alam et al. (2018) was used in this study. Briefly, for each cultivar the total DNA extracts from the leaves were prepared using the CTAB method of Rogers and Bendich (1994). The DNA extracts were then submitted to Diversity Array Technology Pty. Ltd., Canberra, Australia. Each DNA sample was digested with *Pst*I and *Hha*I, then the restriction fragments were ligated to adapters compatible with *Pst*I overhang, followed by PCR-amplification using the DArT-*Pst*I primer (Wenzl et al., 2004). SNPs were detected from the sequencing of the ligated products using an Illumina HiSeq 2000 platform. SNP markers were scored as binary (“1” for presence and “0” for absence) for both reference and alternative alleles. The data were analyzed using DArTsoft14 genotypic data analysis program and DArTdb (Diversity Array Technology Pty. Ltd., Canberra, Australia). The genotypic data of 20 cultivars each of the two extreme phonotypic groups (bulk) that represent susceptible and resistant materials were compared. A bulked sample analysis (Zou et al., 2016) was performed with the DArTseq SNPs of the individuals of both extreme phenotypic groups from the non-biparental population using “QTLseqr” package in the R environment (R Development Core Team, 2013; Mansfeld and Grumet, 2018). SNP indices and delta (Δ) SNP indices were calculated from reference allele and alternative allele frequencies and the read depth of both groups using the following formulas.


$$\text{SNP}\,\text{index}=\frac{\text{Alternative}\,\text{allele}\,\text{frequency}}{\text{Total}\,\text{read}\,\text{depth}}$$

$$△\,\mathrm{SNP}\,\mathrm{index}=\left[\mathrm{SNP}\,{\mathrm{index}}_{\left(\mathrm{susceptible}\,\mathrm{group}\right)}-\mathrm{SNP}\,{\mathrm{index}}_{\left(\mathrm{resistant}\,\mathrm{group}\right)}\right]$$

The QTLseq analysis was performed with a sliding window size of 10^–6^, group size of 20 and 10000 simulations. The cut off for significance of levels Δ SNP index ± 0.3 and LOD ≥ 1.3 were used (Bali et al., 2018; Ramos et al., 2020). Significant markers were identified based on the q-value at the false discovery rate (FDR) of 0.01. To check if these markers were associated with *M. integrifolia* and *M. tetraphylla* species, allele frequencies of these loci were calculated in GenAlEx software (Peakall and Smouse, 2012) using genotypic data of these species, each containing 90 representatives (Supplementary Table 1). To check if the 10 SNP markers identified as associated with AVG resistance are linked to nucleotide-binding site leucine-rich repeats (NBS-LRR) genes, a BLASTN against macadamia genome was performed.

### Selection and Validation of Single Nucleotide Polymorphism Markers

Two out of the 10 SNPs associated with AVG resistance were arbitrarily selected. PCR primers were designed to target the flanking regions of each of the two SNPs (Table 2). PCR was performed with three replicate samples of five cultivars representing the AVG resistant phenotype and 10 cultivars representing AVG susceptible phenotype (Table 1). Each PCR mixture contained 1 × Mango*Taq* reaction buffer (Bioline), 4 mM MgCl_2_, 200 μM of dNTPs, 200 nM of each primer, 2% DMSO, 0.04 μg/μl BSA, 2 Units of Mango*Taq* DNA polymerase (Bioline), 2 μl of DNA template (≤10 ng/μl) and nuclease-free water to a final volume of 50 μl. Thermocycling conditions were an initial denaturation step at 95°C for 5 min, 35 cycles of denaturation at 95°C for 30 s, primer annealing at respective temperature (Table 2) for 30 s and extension at 72°C for 30 s, and a final extension step of 72°C for 5 min. PCR products were electrophoresed in a 1% agarose gel to confirm the presence of expected amplicons. Remaining PCR products were purified using a QIAGEN PCR purification kit according to the manufacturer’s instructions. Purified PCR products were sequenced at the Macrogen Inc, Seoul, South Korea, using the Sanger sequencing method (Sanger et al., 1977). Sequences were trimmed, processed, and aligned using Geneious R10.2.4 software to confirm the nucleotide base positions.

**TABLE 2:** PCR primers used for validation of single nucleotide polymorphism (SNP) markers associated with AVG resistance in macadamia.

Primer name	Sequence (5′–3′)	Annealing temperature (*^o^*C)
SNP1_F	AGTGGTGGAAGTGGATTGTTGC	58
SNP1_R	CTGACAATACCATTCCGCATGA	
SNP7_F	GAGTGGTTGCTATCAACTGC	54
SNP7_R	TAGCACCTGTTGTAGCTTCG	

## Results

### Phenotypic Variability

**FIGURE 1:**
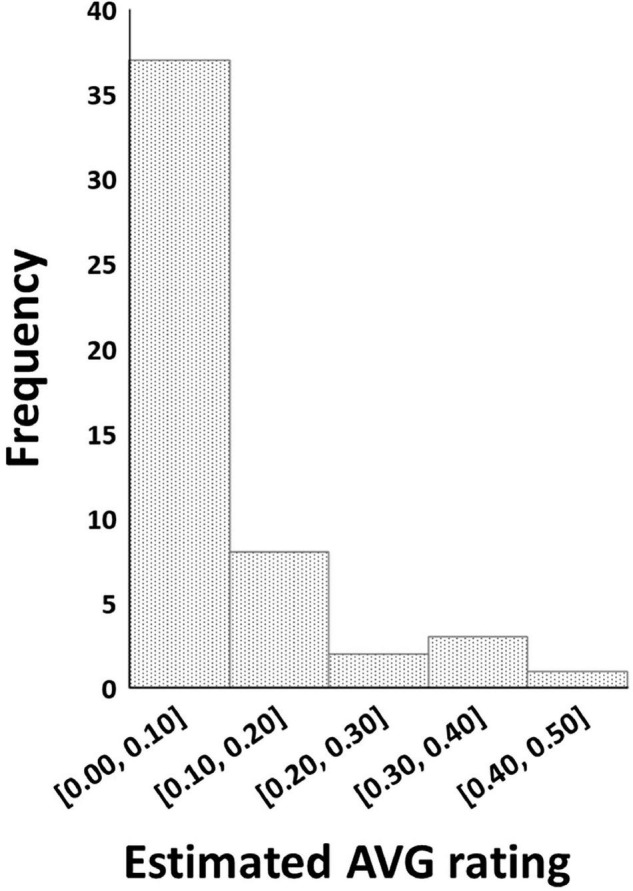
Frequency distribution of macadamia cultivars with different levels of estimated abnormal vertical growth ratings calculated based on Restricted Maximum Likelihood model.

**FIGURE 2:**
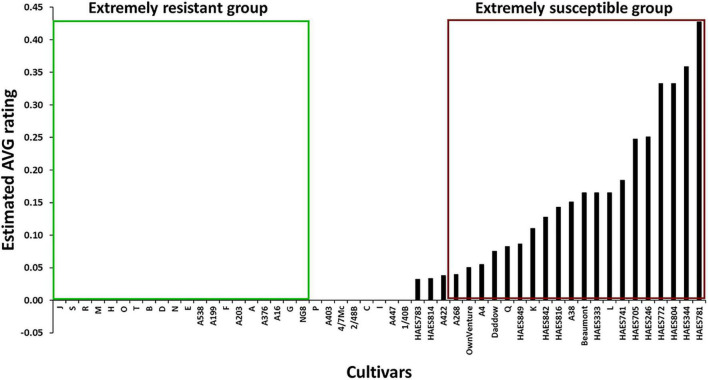
Selection of extreme groups of abnormal vertical growth (AVG)-resistant and -susceptible macadamia cultivars for DArTseq genotyping. Cultivars in the green and red boxes were classified as extremely resistant and extremely susceptible.

The REML analysis showed that the *ER*_*AVG*_ of the cultivars varied between 0 and 0.43 (Wald statistic = 228.61, *P* < 0.001). About 73% (*n* = 37) of the cultivars had an *ER*_*AVG*_ of < 0.1 (Figure 1). Thirty cultivars were in the AVG resistant group (*ER*_*AVG*_ = 0) while 21 cultivars were in the AVG susceptible group (Table 1). Most of the Australian cultivars were in the AVG resistant group, while the Hawaiian selections were in the AVG susceptible group of *ER*_*AVG*_ ≥ 0.04 (Figure 2). The highest *ER*_*AVG*_ = 0.43 was estimated for ‘HAES 781’, followed by *ER*_*AVG*_ = 0.36 for ‘HAES 344’ (Table 1). The HAES group of selection origin showed the highest mean AVG rating while the AMIB group showed the lowest value (Supplementary Table 2).

### Single Nucleotide Polymorphism Markers Associated With Abnormal Vertical Growth Resistance

**FIGURE 3:**
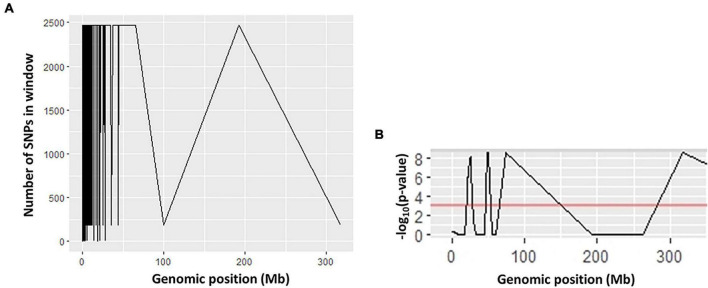
Quantitative trait locus (QTL) statistics plots showing the relationships between **(A)** number of SNPs; **(B)** logarithm of the odds (LOD) and genomic positions.

Ten SNP markers associated with AVG resistance were identified (Table 3). The ΔSNP indices of these markers were >0.300 or <−0.300 (Table 3). Seven of the markers showed negative ΔSNP index values (Table 3). All the 10 SNP markers were highly significant (*P* < 0.001) at an FDR of 0.01. The SNP markers were located in different scaffolds of the macadamia genome. A SNP C > T at the base position 11 in scaffold 13,413 (FLKO01013413.1) showed a Δ SNP index of 0.375, whereas SNP G > C at base position 56 in scaffold 7,350 (FLKO01007350.1) and SNP C > G at base position 18 in scaffold 4,398 (FLKO01004398.1) had Δ SNP index of −0.400 (Table 3). The smoothed SNP/window distribution showed some regions of the genome have extremely low SNP density. A significant decline in the SNP density was observed in the 70–100 Mb and 190–320 Mb genomic positions (Figure 3A). The number of SNPs at the genomic position of 70 Mb was approximately 2,475, but at the 100 Mb genomic position the number drastically lower at about 200 SNPs (Figure 3A). Significant SNP markers associated with the AVG resistance showed logarithm of odds (LOD) values >8 in the genomic position of approximately 25–60 Mb (Figure 3B) indicating that they are associated with different genomic regions. Two SNPs (SNP 7 and 10) at chromosome positions 50083 and 26136, respectively, showed clear distinction between *M. integrifolia* and *M. tetraphylla* wild germplasm (Table 4). Alternative allele frequency of the SNP 10 (a bi-allelic locus) was four-fold higher in *M. tetraphylla* than in *M. integrifolia* (Table 4). BLAST results revealed that none of the markers was linked to NBS-LRR genes or any hormone regulatory genes.

**TABLE 3:** Single nucleotide polymorphism (SNP) markers associated with AVG resistance in macadamia.

SNP No.	Scaffold	Chromosome position	SNP	Base position	Δ SNP index	*P* value	*Q* value
1	13413	21838	C > T	11	0.375	1.35E-06	7.09E-05
2	3285	22060	C > G	17	0.350	9.85E-07	5.53E-05
3	2372	24922	A > G	59	0.300	1.32E-08	3.71E-06
4	2	74827	G > C	47	–0.300	0	0
5	2240	21340	G > A	38	–0.300	8.16E-06	0.0004
6	2	23420	G > T	51	–0.350	1.36E-07	1.68E-05
7	706	50083	C > A	42	–0.375	0	0
8	7350	24050	C > T	15	–0.375	5.20E-08	8.39E-06
9	7350	24050	G > C	56	–0.400	5.20E-08	8.39E-06
10	4398	26136	C > G	18	–0.400	7.56E-09	2.73E-06

*Δ SNP index – delta SNP index was calculated by subtracting the SNP index of extremely resistant group from the SNP index of extremely susceptible group. P and Q (optimized for false discovery rate) values based on bulked samples analysis using QTLseq procedure.*

**TABLE 4:** Frequencies of reference and alternative alleles at ten SNP loci significantly associated with AVG resistance trait in *Macadamia integrifolia* and *M. tetraphylla* wild germplasm.

SNP no.	Chromosome position	Allele frequency (%)			
		*M. integrifolia*		*M. tetraphylla*	
		Reference allele	Alternative allele	Reference allele	Alternative allele
1	21838	92	8	100	0
2	22060	98	2	100	0
3	24922	100	0	96	4
4	74827	100	0	50	50
5	21340	86	14	100	0
6	23420	100	0	99	1
7	50083	13	87	80	20
8	24050	71	29	99	1
9	24050	100	0	100	0
10	26136	75	25	5	95

### Single Nucleotide Polymorphism Sequences and Validation of Selected Single Nucleotide Polymorphism Markers

**FIGURE 4:**
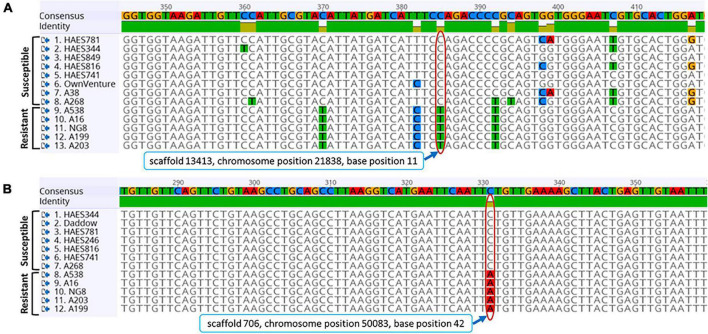
Single nucleotide polymorphism (SNP) locus **(A)** C > T on scaffold 13,413 and **(B)** C > A on scaffold 706 of macadamia cultivars amplified by SNP1_F/SNP1_R and SNP7_F/SNP7_R primer pairs, respectively. Resistant and Susceptible indicates cultivars that show abnormal vertical growth (AVG)-resistance or AVG-susceptibility. Red elongated circles indicate base positions in both loci and their position details are given in rectangle below the sequences of SNP. Color codes of nucleotides A, C, G, and T were red, blue, orange, and green, respectively. The numbers on the consensus sequences show the position of nucleotides. Sequence of each cultivar was from multiple replicates.

The sequences for all 10 SNPs are given in Supplementary Table 3. The two arbitrarily selected SNPs (SNP1 and SNP7) were validated. A sequence with a 715 bp size that carried an SNP on scaffold 13,413 was amplified by primers SNP1_F/SNP1_R. This SNP had a “C” allele at base position 11 in susceptible cultivars (‘HAES 344’, ‘HAES 781’, ‘HAES 849’, ‘HAES 816’, ‘HAES 741’, ‘Own Venture’ and ‘A38’) rather than “T” allele of resistant cultivars (Figure 4A). The primer pair SNP7_F/SNP7_R amplified an 842 bp fragment on scaffold 706 of macadamia genome (FLKO00000000) that contained a SNP with a predicted C > A base at position 42. Susceptible cultivars that included the Hawaiian cultivars (‘HAES 344’, ‘HAES 741’ and ‘HAES 781’), and Australian cultivars (‘Daddow’ and ‘A268’) had the “C” allele at this SNP rather than the A allele of AVG-resistant cultivars of ‘A538’, ‘A16’ and ‘NG8’ (Figure 4B). In each of these loci, multiple individuals of same cultivars had the same allele suggesting that both groups (resistant and susceptible) are homozygotes for the alleles. The cultivar ‘A268’ that was determined to be moderately susceptible to AVG also possessed alleles for susceptibility in the two SNPs tested (Figure 4).

## Discussion

This study predicted 10 SNP markers with significant link to AVG resistance. Further sequencing analysis of two arbitrarily selected SNPs validated macadamia varietal susceptibility to AVG and revealed that hybrids with a high percentage of *M. tetraphylla* in their genomes were more resistant to AVG than those that were mainly *M. integrifolia*, suggesting the resistance-associated alleles may be linked to the former. A few loci associated with AVG resistance appear to be derived from the genomic introgression of *M. tetraphylla* through interspecific hybridization. AVG resistance is not based on the origin of selection but the species of the macadamia cultivars.

Generally, Hawaiian cultivars were highly susceptible, while Australian cultivars showed moderate to high resistance. A few hybrid cultivars such as ‘A4’, ‘A268’ and ‘Beaumont’ that contain 10 – 50% of *M. tetraphylla* genome (Peace et al., 2001b) showed susceptibility to AVG. Macadamia cultivars ‘A16’, ‘NG8’, ‘A199’ and ‘A538’ that exhibited resistant reaction to AVG possess 30 – 50% of *M. tetraphylla* genome (Peace et al., 2017). In contrast, the cultivars ‘HAES 781’, ‘HAES 344’, ‘HAES 804’, ‘HAES 741’, ‘HAES 816’, ‘A38’, ‘Daddow’ and ‘Own Venture’ that showed susceptibility to AVG contain 100% *M. integrifolia* genome (Peace et al., 2017) and belong to the gene pools 1 - 4 (Peace et al., 2001b). Our study revealed that most of the cultivars from the Australian macadamia industry breeding (AMIB) program were resistant to AVG; more importantly all the recent releases (Russell, 2018) including “G,” “J,” “P” and “R” that were highly resistant. ‘Daddow’, ‘Own Venture’ and ‘Beaumont’ represent Australian early selections (AES) that were obtained from the 1952 seedling surveys in Australia (Alam et al., 2018). Australian ‘A’ series cultivars such as ‘A4’, ‘A16’, ‘A38’, ‘A199’, ‘A203’, ‘A268’ and ‘A538’ were released as open-pollinated hybrids between Hawaiian and Australian cultivars by Hidden Valley Plantation (HVP) (Peace et al., 2002; Alam et al., 2018). In this study, we found that most of the ‘A’ series cultivars such as ‘A538’, ‘A199’, ‘A203’ and ‘A16’, except the cultivars ‘A38’, ‘A4’ and ‘A268’ were highly resistant to AVG. This study showed that the cultivar ‘A268’ had alleles for susceptibility in eight out of 10 SNPs associated with AVG resistance, indicating AVG susceptibility in the two SNPs sequenced, and exhibited moderate susceptibility or partial resistance to AVG. The AVG-susceptible cultivar ‘Beaumont’ belongs to gene pool 5 as does ‘A268’ (Peace et al., 2001b).

One limitation of this study is that there is currently no complete assembled macadamia genome, hence, our markers could not be easily mapped. However, our 10 SNP markers were presented at scaffold level (Table 3). Recently, Langdon et al. (2020) generated multiple maps of macadamia using small number of markers, and Nock et al. (2016) sequenced ≈79% of the macadamia genome. When a more complete reference genome becomes available, the positions of the markers on macadamia chromosomes can be determined. Only two arbitrarily selected SNPs were validated, validation of all the 10 SNPs in all 40 or more macadamia cultivars will strengthen the use of the SNPs in MAS for AVG resistance. Assays such as Taqman or KASP assays may be developed and run for each SNP to reveal both alleles for each individual to confirm its genotype.

The macadamia accessions used in this study represented a mixture of commercial cultivars with field resistance and susceptibility to AVG. A large number of macadamia trees were phenotyped for AVG using a simplified 0–3 severity rating scale based on an established protocol (Akinsanmi, 2017). This limitation of the few rating scale may possibly miss small-effect trait loci associated with AVG resistance. The pooled analysis used in this study was based on the two contrasting groups regardless of population structure (Xu et al., 2008; Sun et al., 2010) and not from a biparental segregating population. In this study, we used 51 macadamia cultivars, which is effective for identifying large-effect trait loci, but a large number of genotypes is required to identify both small- and large-effect loci and for a genome wide association study. Although we did not include population structure in the model, the results revealed that most of the cultivars of *M. integrifolia* origin were susceptible to AVG, whereas *M. tetraphylla* cultivars were resistant. Further genomic investigation of wild and cultivated germplasm may explain the origin of AVG resistance. It is imperative to look at the inheritance patterns of resistance to AVG and to develop more closely linked genetic markers for the resistance. To achieve this, controlled crosses should be established between susceptible and resistant cultivars. When the pathogenic agent that causes AVG is identified, it will offer an opportunity to phenotype progeny rather than relying on natural field infection, which may take over 10 years for symptoms to be expressed.

In conclusion, this study identified the extent of variability in AVG resistance in 51 macadamia cultivars representing several groups. A genomic investigation revealed 10 SNPs associated with AVG resistance/susceptibility trait. Variability in Hawaiian and Australian cultivars provided evidence of resistant alleles in Australian germplasm, while suggesting that a few markers associated with the trait appear to be derived from *M. tetraphylla* via interspecific hybridization. Therefore, there is an opportunity to develop AVG resistant cultivars through breeding. While appropriate for the analysis, the method used in this study was constrained by limited number of cultivars representing each gene pool. Although preliminary in nature, this study provides new insights into the resistance present in macadamia cultivars and its associated SNP markers that would be useful for screening macadamia germplasm for AVG resistance. Identification of QTLs showing the signature of AVG resistance, and their associated genes, could be the focus of future studies.

## Data Availability Statement

The original contributions presented in the study are included in the article/Supplementary Material, further inquiries can be directed to the corresponding authors.

## Author Contributions

MZ, OA, and AG conceived the idea. OA received research grants for the project, performed phenotyping, the lead researcher and principal research project supervision. MZ and MA did phenotypic data analysis. BT and MA provided the genotypic data. MZ performed genotypic data analysis, molecular experiments, and drafted the manuscript. All authors edited, read and approved the final manuscript.

## Conflict of Interest

The authors declare that the research was conducted in the absence of any commercial or financial relationships that could be construed as a potential conflict of interest.

## Publisher’s Note

All claims expressed in this article are solely those of the authors and do not necessarily represent those of their affiliated organizations, or those of the publisher, the editors and the reviewers. Any product that may be evaluated in this article, or claim that may be made by its manufacturer, is not guaranteed or endorsed by the publisher.
