# Supplementary material for: Discovery of Single Nucleotide Polymorphisms for Resistance to Abnormal Vertical Growth in Macadamia
Source: Front Plant Sci. 2021 Dec 24;12:756815. doi: 10.3389/fpls.2021.756815 (PMC8739493; doi:10.3389/fpls.2021.756815)
Supplement: Supplementary file 2 [file Table_2.DOCX]

**Supplementary Table 2** | Mean AVG ratings of cultivars from four different groups of origins indicated the dominance of Hawaiian cultivars (HAES) on AVG susceptibility.

| **Cultivar origin** | **Mean AVG rating** |
| --- | --- |
| AES | 0.075 |
| AMIB | 0.016 |
| HAES | 0.209 |
| HVP | 0.025 |

AES: Australian Early Selection; AMIB: Australian Macadamia Industry Breeding; HAES: Hawaii Agricultural Experiment Station; HVP: Hidden Valley Plantation.
