# Supplementary material for: Discovery of Single Nucleotide Polymorphisms for Resistance to Abnormal Vertical Growth in Macadamia
Source: Front Plant Sci. 2021 Dec 24;12:756815. doi: 10.3389/fpls.2021.756815 (PMC8739493; doi:10.3389/fpls.2021.756815)
Supplement: Supplementary file 3 [file Table_3.DOCX]

**Supplementary Table 3** | Sequences of the ten SNPs associated with AVG resistance trait. The position of SNP is marked using square brackets and forward slash (/) indicates the two possible alleles in each sequence.

| **SNP No.** | **Sequence** |
| --- | --- |
| 1 | TGATCATCTC[C/T]AGACCCTGCAGTGGTGGGAATCGTGCACTGGATGCATCTAGGGAAACTTATTTAGGCA |
| 2 | TGCAGCATATAATGTG[C/G]CTTGCATAGGCTAATATTAGAAAGCCAATAAAAAAAAGGAAGTCTAAGGTAC |
| 3 | TGCAGGCTGGTGCTTTGCTTGCCGATGAAGATGTCGACGAAAACACACCATCGAGGAG[A/G]ACCTCTACTT |
| 4 | TGCAGACAATATGCATGGGATTAGCCGCTTCAATGGGATCTTTTAT[G/C]GTGGTCGGAGGAGAAATTACCA |
| 5 | TGCAGAGGACACGGTCTCCAAGTTTCCTATTTCTCAG[G/A]GAGCACATGCATTCCTTCGAGGTTTCCCTAT |
| 6 | TGCAGGGGCATCCTGTATTGCTGAATAGAGCACCCACTTTGCATAGATTA[G/T]GCATCCAGGCATTCCAAC |
| 7 | CAGTTCTGTAAGCCTGCAGCCTTAAGGTCATGAATTCAATT[C/A]TGTTGAAAAGCTTACTGAGTTGTAATT |
| 8 | GCAGTTATAATCAA[C/T]GCATTTCAGGTAAGAAGGGCATTTGAAATATGGGTTCTGTGTCAATCACCTTT |
| 9 | GCAGTTATAATCAACGCATTTCAGGTAAGAAGGGCATTTGAAATATGGGTTCTGT[G/C]TCAATCACCTTT |
| 10 | TGCAGAAGATACCGAAG[C/G]CCCTTGCATTGACAAAAAAATCTTCGATGTCGGTGGTGATTTCGACTTCAA |
